# Nursing staff's actions during older residents’ transition into long-term care facility in a nursing home in rural Norway

**DOI:** 10.3402/qhw.v9.24105

**Published:** 2014-10-08

**Authors:** Marianne Eika, Geir Arild Espnes, Sigrun Hvalvik

**Affiliations:** 1 Department of Social Work and Health Science, Faculty of Social Sciences and Technology Management, Research Center for Health Promotion and resources Hist-NTNU, NTNU, Trondheim, Norway; 2 Department of Social Work and Health Science, Faculty of Health and Social Studies, Telemark University College, Porsgrunn, Norway; 3 Center for Caring Research—Southern Norway, Grimstad, Norway

**Keywords:** Residential long-term care, nursing staff, admission, rural community, context

## Abstract

Working in long-term care units poses particular staff challenges as these facilities are expected to provide services for seriously ill residents and give help in a homelike atmosphere. Licensed and unlicensed personnel work together in these surroundings, and their contributions may ease or inhibit a smooth transition for recently admitted residents. The aim of the study was to describe and explore different nursing staff's actions during the initial transition period for older people into a long-term care facility. Participant observation periods were undertaken following staff during 10 new residents’ admissions and their first week in the facility. In addition 16 interviews of different staff categories and reading of written documents were carried out. The findings show great variations of the staff's actions during the older residents’ initial transition period. Characteristics of their actions were (1) in the preparation period: “actions of sharing, sorting out, and ignoring information”; (2) on admission day: “actions of involvement and ignorance”; and (3) in the initial period: “targeted and random actions,” “actions influenced by embedded knowledge,” and “actions influenced by local transparency.”

The transition into a long-term care facility (LTCF) can be traumatic for residents and their family members, and demanding for staff (Davies, 2005). Many older residents may have experienced multiple and simultaneous transitions (Young, 1990, Aksøy, 2012, Eika, Espnes, Söderhamn, & Hvalvik, 2014) before they finally move into an LTCF. Staff in LTCFs provide services for residents with complex chronic and acute medical problems and give daily care in a homelike atmosphere (Hauge, 2004, Ryvicker, 2011). Most Norwegian nursing homes are run by the municipality, and are close to the home communities of the residents (Jacobsen & Mekki, 2011). There are no formal staffing standards (Harrington et al., 2012), and Jacobsen and Mekki (2011) claim the staff coverage in Norway is more than double that of most European countries and that staff are relatively formally qualified.

Transition theory in nursing has been defined differently during the development of the theory, and has varied with the context in which the term has been used (Kralik, Visentin, & van Loon, 2006). According to Chick and Meleis (1986), transition is understood as a passage from one life phase, condition, or status to another. It refers to both process and outcome of complex person–environment interactions, and may bring about fundamental changes in the person's view of self and the world (Meleis & Trangenstein, 1994). Transitions are initiated by a change or marker event that brings about disequilibrium and upheaval that requires new patterns of response. Transitions are processes that may take time (Schumacher, Jones, & Meleis, 1999), and it is important that older people who may experience multiple transitions in late life, are given the time to “experiment with different strategies and patterns of responses and to incorporate them into one's own repertoire” (p. 130). Strategies may involve the development of new roles, new relationships, and new skills. Transition theory in nursing highlights the importance of professional support in these periods of change. Facilitating healthy transition processes and outcomes focus on nursing therapeutics and process indicators which allow early assessment and interventions (Meleis, Sawyer, Im, Hilfinger Messias, & Schumacher 2000).

Our study is concerned with staff's actions in the initial transition period into LTCF which is a critical time in the older residents’ transition experience (Young, 1990, Reed, & Morgan, 1999). Research examining staffs’ perspectives during older residents’ transition into LTCF is scarce (Wiersma, 2010, Ryan & McKenna, 2013), and we have been unable to find research describing different staff's actions targeted at this group. However, various aspects of staff involvement are presented through the lenses of family members (Hertzberg, Ekman, & Axelson, 2001, Davies, 2005, Flynn Reuss, Dupuis, & Whitefield, 2005) and through the residents’ perspectives (Kahn, 1999, Heliker, & Scholler-Jaquish 2006, Coughlan & Ward, 2007). Yet it is also important to focus directly on staff's actions to identify potential factors that may ease or inhibit a smooth transition for recently admitted residents.

## Aim

The aim of the study was to describe and explore nursing staff's actions during older people's transition into LTCF in a Norwegian rural context.

## Methodology

The epistemological position was constructivist hermeneutical. The constructivist position is an analytic middle ground between reality and representation (Gubrium & Holstein, 1997). Horizon is a central concept in the tradition of Gadamer (2007), and a metaphor for how we perceive and interpret reality. Prejudices as part of our horizons are prerequisite for interpretations (Debesay, Nåden, & Slettebø, 2008), and identification of the researchers’ pre-understandings is part of exceeding one's horizon (Gadamer, 2007). An important part of the researchers’ pre-understandings is connected to their background as registered nurses and researchers with particular interest in the care of older people.

## Setting and participants

The municipal nursing home is situated in rural southern Norway. The LTCF is split into three units (LTCU) each with 10 private rooms. The facility is organized according to a modified primary nursing model (Lakso & Routasalo, 2001) where each nurse is primary contact for five residents, and the auxiliaries are secondary contacts for three residents each. Staffing ratios varied with the shifts. During daytime weekdays the staffing levels in each unit were three staff to 10 residents. At the evening shifts there were usually two staff, auxiliaries, or assistants in each unit and one nurse in charge of the 30 residents in the facility. The assistants do not have the responsibility for any particular residents, and are used where needed across the units. In Norway the nurses have 3 years nursing education from university college, the auxiliaries have 1 or 2 years nursing education from high school (Høst, 2010), and the assistants have no health care education apart from short introductory courses at the workplace.

In our study the nurses and auxiliaries interviewed were between the ages of 30 and 60. Nine were female and two were male. The assistants were females between the ages of 20 and 30. The nurses and auxiliaries had long work experience with older people, whereas the assistants had less than 5 years.

## Data collection

Three approaches – periodic participant observations, semistructured interviews, and reading of relevant documents were used to collect data. All the data were recorded in field notes and verbatim transcript. The transcribed texts from interviews and the texts from field notes were regarded as texts equally important (Atkinson & Coffey, 2002), and contributed to rich descriptions.

Participant observations were undertaken periodically following 10 new residents on admission day and their first week in the LTC unit. Observations were carried out in the residents’ private rooms, the corridors, the living room, the dining room, the kitchen, and the staff's room. First author spent on average 4 h a day and was in the facility at different times, and during weekends and summer holiday. A total of 200 h of observations were undertaken periodically in the 8-month period, from June 2011 to January 2012. The design of the study with participant observations involved not only the nursing staff, but also the residents, and therefore gave insights into their experiences too.

Field notes recorded staff activities with the newly admitted resident and each other on admission day and the initial period after the admission. Also the field notes included researcher's feelings and reactions which assisted in realizing the researcher's prejudices brought to the study.

In addition, interviews were conducted with four nurses, one head nurse, six auxiliaries, and five assistants. Recruitment was by voluntary participation and snowball effect. Before the project started staff members were informed both orally and in writing by first author and later by head nurse about the project. Written information was put on the wall in the head nurse's office where staff could write their names if they wanted to participate.

The interviews took place in a small room in the nursing home outside of the LTCF, and lasted about 1 h. The interviews were semistructured with an interview guide focusing on the preparation period before a new resident arrived, the admission day, and the initial period after placements, with particular focus on residents’ self-care capabilities. Follow-up on what the respondents themselves elaborated upon was attempted.

The text should be: Documents read were residents' charts and medical histories at the time of arrival as were the daily care plans on the computer and in the residents' bathrooms. These were mainly consulted to fill in on the data from observations and interviews (Fangen, 2010).

## Data analysis

The data collection and analysis occurred concurrently. This meant that analytical reflections started automatically and opened up for the pursuit of new perspectives and hunches as the field work and interviews went along (Sandelowski, 2000, Vike, 2003). The analysis continued in a more systematic way when the researcher immersed herself in the material to gain a sense of the whole, and used the analysis methods outlined by Foreman and Damschroder (2008) and Graneheim and Lundman (2004) as the major guidelines for the systematic analysis of the data. This was done in different ways. Transcripts, field notes, and written documentation were read several times. Additional listening to the audio files, and writing down comments, associations, and memos between and across interview transcripts and field notes were carried out. Memos served to initiate the data analysis by identifying and sharpening categories and themes that began to emerge. In the reduction phase, a systematic approach to the data was developed aimed at focusing on relevant data to answer the research question. Meaning units and codes representing topics, concepts, or categories of events did this. These codes were then grouped together to create subcategories and categories which were then arranged into themes (Table I).

**Table I:** Themes and categories regarding staff's actions during older people's transition into long-term care facility (LTCF).

Time—chronological order	Themes	Categories
Preparation period	Actions of sharing, sorting out and ignoring information	Dealing with -Competing tasks -Uncertainty -Routines
Admission day	Actions of involvement and ignorance	Variations in how new residents are met Residents’ previous residence matter
Initial period	Targeted and random actions	Contextual features Self-care activities Medical Treatment
	Actions influenced by embedded knowledge	There is always someone in need of an LTCF placement The LTCF—a home taken-for-granted Mentally lucid residents manage on-their-own
	Actions influenced by local transparency	Familiar people and places Good or dubious reputation

## Rigor

Rigor was secured by the 8-month time frame of the study and the different approaches investigating the same phenomenon (O'Reilly, 2012). The time frame and the periodic participant observation periods paired with interviews allowed the researcher to ask new questions and follow new direction as the fieldwork went along (Vike, 2003). Data collection from different sources gave rich material, allowing complexity and in-depth observations to occur. Also the use of an experienced qualitative researcher at several points in the analysis was schemed to reduce bias.

## Ethical considerations

The research was executed in accordance with the Declaration of Helsinki. Approval was obtained from the Regional Committees for Medical and Health Research Ethics in Norway (2011/153b). Formal access to the field was made through the municipal health care authorities. The staff participants were assured confidentiality. Their participation was voluntary, and they were informed they had the right to withdraw at any time without stating a reason. Before data collection, written informed consent was obtained from all staff participating in the interviews.

Residents were asked on arrival day if they accepted that first author participated in their daily care the first week after arrival. Eight residents accepted. For two residents their families consented on their behalf. All 10 residents were orally informed about the project.

## Findings

In what follows the themes identified are presented in chronologic order starting with the preparation period, the admission day, and the initial period. The theme identified in the preparation period was “actions of sharing, sorting out, and ignoring information.” The theme identified on admission day was “actions of involvement and ignorance.” The initial phase themes identified were “targeted and random actions,” “actions influenced by embedded knowledge,” and “actions influenced by local transparency.”

The themes are not mutually exclusive, and intertwine in complex ways. In addition to quotes, abstract narratives are used to illustrate the influence staff's action had on the resident.

### Preparation period

The preparation period was characterized by staff actions that varied from sharing, sorting out, and ignoring information.

### Actions of sharing, sorting out, and ignoring information

In this period the staff had to deal with competing tasks, uncertainty, and routines.

#### Dealing with competing tasks

Most staff started preparing themselves for the arrival of a new resident on admission day. This, paired with the other daily tasks, gave them little time to prepare. The time span between the death of a resident and the arrival of a new resident varied between 2 and 6 days. The admission team decided who would get a LTC-placement, and they provided the head nurse with some written information about the prospective resident which she shared with all staff present at the joint morning shift report on admission day.

The prospective resident could arrive from hospital, community dwellings, home, or from other facilities within the nursing home. The exact time of arrival was often not known, however, if the residents arrived from hospital, staff knew they would probably arrive in the afternoon at the time of the shift report.

Moreover, written information about the resident-to-be was sometimes ignored by staff. During the summer holiday which was considered a strenuous time to work because of all the supply staff, none of the staff appeared to read the information about the expected resident “Ann” before arrival. When she arrived with her family member in the morning, she was met by the nurse. On the threshold to her room the nurse said “look, your name is on the door so we are expecting you, and look at the beautiful flowers on the photo.” “Ann” replied in a hardly audible voice that she could not see it.

The nurse appeared unaware of the resident's impaired vision. During the first week her low vision seemed to be disregarded by most staff in this unit. The primary auxiliary told on the last day of “Ann's” first week that she had not yet read the information about her.

#### Dealing with uncertainty

Some nurses were concerned about maintaining continuity across health care settings particularly regarding resident's medical condition and medication. Yet information from hospital could be lacking on arrival day. Then using the phone was the most appropriate means to get information. Still, using the phone could be frustrating because sometimes nobody answered or the person did not know the resident in question.

Furthermore the head nurse experienced that sometimes the resident was welcomed ad hoc:

I write in the program book the expected resident's name and the room. I realize that sometimes I can be more specific about who is to welcome the new resident. If there is a nurse in the unit it is self-evident she will do it, but if there is none then sometimes it is random that the one who accidentally meets the new resident welcomes. This is not optimal, I have thought about it and we need to do something about it. Even though I have been in the game for many years, it is weird that such simple things—it takes such a long time before they are written. But it has something to do with balancing how much am I to interfere and how much are they to think themselves (interview head nurse).

The head nurse had a balanced ideology on behalf of her staff that all staff knew and would flex between following procedures and their skills there and then in interaction with the new resident. Not all staff appeared to be familiar with this, however, and information in the preparation period could be ignored or lacking. The assistants were expected to take responsibility for the prospective resident's room on equal footing as the auxiliaries. Still this expectation was not always carried out: “Sometimes the information is poor so you have to take some initiative yourself, and I am unsure if the assistants do that” (interview assistant).

#### Dealing with routines

The different staff had different procedures to follow in this period. The primary nurse in the unit had the responsibility to check the information available and have a preliminary overview of the resident's condition and needs, whereas the auxiliaries and the assistants mainly had the responsibility to prepare a tidy, clean, and welcoming room, in addition to taking care of the residents. The head nurse made sure that the resident's name was on the door before arrival.

However, routines were not always carried out by the staff, such as preparing the resident's room properly, which puzzled the staff nurse:

I expect such matters to be automatic, but I have experienced that they are not, so maybe it is in its place to have more routines, that I have to give more specific information in the program book. At the same time I find that a little unnecessary—when we are organized according to primary nursing and each claim they want to be responsible. But it is sort of divided here—so I have, I check the room the day the new resident will arrive (interview head nurse).

### Admission day

Admission day was characterized by actions that differed from involvement to ignorance.

### Actions of involvement and ignorance

In these periods the new residents’ previous residence appeared to matter, and it varied as to how staff met the new residents.

All staff perceived admission day as important and the head nurse prepared the grounds for this: “It is vital for the new residents to feel expected and that their arrival is prepared because this is going to be their new home for the rest of their lives” (interview head nurse). Most staff had a nuanced understanding of the older resident in transition into LTCU. Some underscored the residents’ experiences of losses, such as losses of belongings, losses of mastery of activities of daily living, and the grief and sadness of losing their previous lifestyle. However, they also experienced that residents were pleased with this arrangement and felt safe, less lonely, and were relieved they were no longer a burden to their family members. Furthermore, for some their health improved.

#### The resident's previous residence matter

These understandings of the new resident paired with circumstances such as the resident's previous residence appeared to influence staff's actions. If a resident was transferred between facilities in the nursing home, the staff found a convenient transfer time for the staff in both facilities. They assumed, as one of the nurses claimed, that the resident was already familiar with the routines, the joint living room area, and living together with other people. If a resident arrived from hospital, however, the time of arrival in the afternoon was inconvenient, and particularly the nurses were frustrated because they had less time to welcome the resident and his family member(s) properly. Sometimes a day shift nurse worked overtime to welcome the resident whereas at other times the resident was welcomed by the nurse after the afternoon shift report was over, or by an auxiliary.

Moreover if a resident arrived directly from home, concrete arrangement of arrival time could be made, illustrated by the following: The nurse had phoned the prospective resident at home 2 days ahead of the admission and arranged for her to arrive in the morning so that she had time to welcome her properly. On arrival day “Helga” arrived alone in her electric wheelchair and was met by the primary nurse who showed her the room. There the nurse carried out the expected procedures at the same time as she listened attentively to the new resident's wishes and worries. The nurse signaled she had time for the resident, and the pace and rhythm in their interaction and dialogues appeared to give the new resident time to respond and take the initiative. Furthermore the nurse prepared the resident for lunch and the other residents at the table where she was going to sit. Also she encouraged “Helga” to contact staff at any time.

This admission was well planned and appeared to be a good start for the resident's further stay in the unit. However, “Helga” had to arrive on her own without her child who was unable to rearrange the work schedule due to the short notice.

#### Variations in how new residents are met

As a rule the nurse in the unit welcomed the new resident. Apart from following the checklists and procedures the nurses appeared to welcome in slightly different ways. If the nurse was absent on arrival day it varied to a greater extent how the resident was met. The auxiliaries had little practice in welcoming new residents which influenced their approaches; some were confident and appeared to follow the procedures in roughly the same way as a nurse, whereas others seemed uncomfortable with this role. Also some appeared unaware of what they were expected to do. In the middle of what they were doing one could hear “oh, yes, is a resident coming? I had forgotten — which room” (interview nurse). Both staff categories were influenced by the daily routines and the needs of the residents already there. Some claimed it was difficult to focus on the new resident while thinking about all the other tasks that needed to be done.

### Initial period

The initial period comprised mainly the first week after the resident's arrival. Three main themes were identified; “targeted and random actions,” “actions influenced by embedded knowledge,” and “actions influenced by local transparency.”

### Targeted and random actions

In the current study targeted and random actions could be associated with contextual features, and staff's attendance to the residents’ self-care and medical treatment.

#### Contextual features

The predominant oral culture influenced staff actions. Many believed the demand for continuous updated written information was meaningless: “… who do we write for; nobody reads” (interview nurse). Especially full-time auxiliaries claimed they had the overview in the small units and knew what they needed to know about the new residents without reading about them. However, the nurses working shifts across all units during afternoon and weekend shift sought written information and guidance with colleagues. The major occasion for seeking written information for all staff was when they had been off work some days. Staff worked shifts and many had part-time positions, and depended on ways of getting and giving information about the new resident. At the time of the study no formal staff meetings were arranged to maintain this need. Although the nurses arranged for the other staff to read information from the previous health care setting, not everybody appeared to do so. Furthermore the oral shift reports lasted about five minutes for each unit. However, after the joint morning report, staff in each unit was expected to plan the day's work together. The most important way of information sharing among staff was continuous on-the-spur-of-the-moment staff interactions: “I believe we cooperate well I sort of get something all the time” (interview auxiliary).

The head nurse's participation in the morning shift reports seemed to be central in directing the staff's actions toward new residents: On the fourth morning after “Olav's” arrival the head nurse supplemented the night nurse's report underscoring that “… he is very social, and he does not express how he really feels.” In the unit afterwards, the nurse supervised the two assistants in detail how to interact with the resident in this matter.

#### Self-care activities

Residents’ self-care capabilities were treated differently. A general attitude was that it would take some time before they would know the new resident: “Eh you have to sort of try and fail a little initially; you have to get to know the resident” (interview auxiliary).

Primary contacts were aware of the distress some new residents experienced initially when needing assistance, and discussed with colleagues how to approach them in the best possible way. The routine writing of care plans within 3 days after admission, however, appeared to work against the staff's approach of gradually getting to know the residents. These plans were put on the wall in the resident's bathroom and provided detailed descriptions of the residents’ need for help and what they managed on their own. They were updated once a year before the summer holiday: “Then everything needs to be spick-and-span before the supply staff comes” (interview nurse). Most permanent staff, however, appeared to regard these plans as guidelines only and did what they thought was best as the situations occurred. Although some focused on aspects of the resident's physical self-care maintenance others seemed to automatically take over:

… I was about to wash the resident in the morning and pull off his t-shirt eh and helped him sit on the toilet. While he was sitting there I was tapping water into the sink because I had not done that, and while doing this I saw in the mirror that he pulled off his t-shirt himself, and I, gosh, I had a revelation … yes, he could manage on his own and I acted as if he could not … just a little more time to get started, so that was a little embarrassing (interview auxiliary).

Likewise others admitted they were too quick to help out:

I experienced today, a classical example, and I did not even reflect on it; but I'm in a resident's room together with the auxiliary, and I know that the resident is able to brush her hair. When the other auxiliary is about to give her the brush my hand automatically grabs it and I quickly brush her hair—it may be valuable for the resident herself to be allowed to do that, and then I in my eagerness … (interview nurse).

One nurse found it meaningless to spend a lot of time encouraging frail elder's self-care during the morning care because it was both time consuming for the nurse and exhausting for the resident. He would rather prioritize the resident's participation in some other activity during the day. Yet others experienced that new residents wanted help with everything after admission, and that they had to supervise both resident and family members in this matter.

Especially some assistants seemed to believe that all that self-care meant was that residents washed their face and hands themselves. However, they underscored other aspects of self-care such as respecting the new resident's boundaries initially, and avoiding exposing their intimate body parts during morning care.

Often staff experienced that the information from the previous health care setting was not transferable in the current LTC- setting, and they did it their way. One auxiliary engaged a resident who did not want to do anything herself, and at times her behavior was threatening. Information from the previous health care setting advised that there be two staff during the morning care, and to use plastic utensils at meals because the resident could throw dishes around. Initially there were two staff during the morning care, but after a few days the primary auxiliary changed her strategy:

I went in alone and put the wash bowl in front of her and asked her to try herself. I arrived back after 15 minutes and the resident had done nothing. I said “are you not going to wash yourself?” “Ye-e-e-s” “Yes, but then you have to sit up.” No, she was not able to do that. Then I say “but may I help you up to sit on the bedside?” This is how we started. After a while she washed her upper body herself, and we stopped using plastic utensils (interview auxiliary).

Also some skilled staff expressed a concern for acting quickly after the resident's arrival in order to maintain some of the resident's previous lifestyle like going out before they became too institutionalized.

Holidays and weekends disturbed the weekday rhythm, and many supply staff appeared unaware of the complexities regarding the new residents’ self-care capabilities. The mix of staff was decisive; if only supply staff worked at a shift, the residents were helped too much during meals, for instance.

How new residents were treated during meals varied among staff as well as between the units. Across all the units some interacted in nuanced ways with the new residents, encouraging their mastery of physical as well as psychosocial needs in their current setting. For example, the recently admitted “Olav” was placed next to a childhood friend's husband. This made “Olav” at ease, and provided continuity with his past life.

Sometimes projects and procedures were prioritized at the expense of the new resident. The following episode illustrates this: On her first morning after arrival, at the breakfast table in the dining room, “Ann” wanted her usual oatmeal and milk for breakfast. However she was persuaded by the staff to eat a slice of bread with cheese and honey. They argued it was important that her nutrition be more varied. During the meal she enjoyed her coffee. When she got her walking frame the auxiliary put her arm around her shoulder and complimented her she had done well eating the bread. The resident replied she was not fond of bread.

Even though the resident probably needed to vary her foods, the staff did not appear to pay attention to the resident's first morning in the unit. Furthermore they seemed unaware of the resident's serious condition. At other times, however, staff could ignore following routines. For instance, all new residents’ nutritional status was to be checked 3 days in a row shortly after arrival, but these observations were not always followed up with appropriate actions.

Moreover, in spite of “Helga's” good start, after admission day she seemed not to be prioritized. Even though she had expressed that “the food tastes so much better in the company of others” and needed to improve her nutritional status, she was often helped a little too late for a joint breakfast with coresidents and had to eat dry bread in her own company.

#### Medical treatment

After arrival knowledge about the resident's medical conditions and medication depended on staff's formal position in the facility. The first days after admission, the nurses focused primarily on the resident's medical condition and prepared for the physician's round which was once a week. Because information from other health care settings could be unclear or lacking, the nurses were busy sorting these matters out in addition to other daily tasks they had to perform.

The auxiliaries did not take part in the physician's round, and their medical information acquisition seemed coincidental. This perceived lack of medical knowledge made some unsure if their care and observations were good enough.

Most nurses worried that the new resident would be put on medication too soon after arrival and not be allowed to react “normally” in this turbulent period, but the time aspect seemed to be forgotten by some: “Kari” had arrived the day before and the night nurse reported to the daytime staff that she was in a mess during the night, and “afraid and anxious.” The nurse in charge of that unit immediately said she would discuss this with the physician and look at the resident's medication. One nightshift nurse student reminded them that this was the resident's first night in an unfamiliar setting, and that she probably needed some time to settle in.

### Actions influenced by embedded knowledge

There were some taken-for-granted assumptions that were rarely if ever expressed by staff. These include: there is always someone in need of an LTCF placement; the LTC unit—a home taken-for-granted; and mentally lucid residents manage on-their-own.

#### There is always someone in need of an LTCF placement

There were waiting lists to get a LTC-placement in the LTCF, and a new resident would move into one of the units when there was a vacant bed. This somehow appeared to influence staff's taken-for-granted attitude of the older person being there after admission day, and not considering him or her being in transition: “Daily routines take over; the resident has to adjust to our routines, it is the same procedure as usual” (interview nurse).

#### The LTC unit—a home taken-for-granted

Even though some openly talked about the “unit as home” for the residents, the notion of home seemed to apply to some staff, too.

Some preferred to have their lunches in the unit's living room instead of the staff's canteen. Most staff seemed absorbed in their own matters then and did not pay much attention to the residents present. The mentally lucid new residents quickly learned to keep away from the living room at these times, but not all the new residents were aware of this, and some were stuck: One wheelchair-bound resident unable to speak was ignored during these times. Moreover the new resident “Rut” suffering from mild dementia seemed to enjoy listening to the staff talking. At times she persisted in her attempts to be included in the staff's interaction; sometimes she was included other times she was ignored.

#### Mentally lucid residents manage on-their-own

Cognitively able residents were in danger of being ignored because staff took it for granted they coped either by asking for help or doing it themselves. The following observation illustrates this: “Helga” depended on help to move around. During the first week at midafternoon coffee for residents and staff, she was never invited. She wondered if staff knew she was incapable of managing herself and needing help to move from her chair into her wheelchair. She expressed modesty in this matter and would have appreciated staff seeing her and inviting her to join them.

### Actions influenced by local transparency

Local transparency could be associated with familiar people and places, and good or dubious reputation.

#### Familiar people and places

The rural community and local staff appeared to influence the cultures in the units. Many female local staff had lived most of their lives in this area, although some had moved to this area from other places in Norway or abroad. The interviews revealed that the management wanted the nursing home to have a good reputation in the community, and appreciated devoted staff. Jobs for auxiliaries and assistants were scarce, and the management could select those most suitable.

Six of the 10 new residents moved into the same unit where most residents were cognitively able with not too demanding physical needs for staff to assist with. Local staff and many residents knew of each other in these surroundings and this created familiarity and connections. At meals staff and residents shared joint knowledge about people and places, and the new residents were quickly included and involved. One new resident, homebound the last 20 years, enjoyed these meals, and was impressed by what her coresidents knew. Another resident transferred from a dementia-specific unit was waited upon by some of the other residents and became more social.

#### Good or dubious reputation

Familiarity with residents contributed sometimes to blur the exchange of information: During the afternoon shift report, the assistant reported that the new resident “Ann” who arrived that morning suffered from cancer with metastases to several organs. One of the auxiliaries immediately said: “I know her. She is the one who worked for years in x institution; she is a very nice lady” (end of report for this resident).

If a resident had a dubious reputation, he or she at times was in danger of being ignored by some staff: At a quiet time before the evening meal, the new resident “Berth” approached one of her previous neighbors, auxiliary X who was sitting in a couch chatting with a colleague. “Berth” asked about X's children. She answered, but did not ask any questions back and redirected her attention to her colleague. This auxiliary however, acted professionally toward this resident in the morning care situations but appeared not wanting to get too personally involved with her.

Word of mouth went within the staff group that a new resident was fond of the ladies. When he asked for help to put on his sweater the auxiliary argued he could do it himself. However, he suffered from a serious illness which periodically made him depend on more help than usual.

## Discussion

Lack of cooperation between and within institutional settings influenced the different staff's actions in multiple ways. Moreover the findings disclose an array of actions in a complex organization where individual staff were influenced by and influenced back on the environment.

From the nurses’ perspective, lack of information about the new resident seemed to pose challenges, particularly concerning medication. Bollig, Ester, and Landro (2010) found that every fifth resident, at the time of their arrival in the nursing home, had misleading information about medication. Because written information could be lacking or be incomplete, the nurses compensated by relying on oral sources of information. Getting information through phone may be problematic. Oral language in this setting placed the sole responsibility for the interpretation on the receiver who would pass this on to colleagues. Spoken words are gone immediately after they are articulated and thereby less reliable than written words (Ong, 1982, 2002). What is written can be read by many and functions as memory and proof for what is to be done and what has been done. Yet communication by phone also made it possible to ask questions about matters that were not so clear in the written information. Furthermore, these activities could be time consuming initially and maybe steal time from direct interaction with the new resident and colleagues. Our findings suggest there is a connection between the auxiliaries’ and assistants’ perceived lack of medical knowledge, and how they assisted and cared for the new residents. The auxiliaries handled their frustration differently.

Although some asked the nurses directly for guidance, others expected the nurse to take the initiative, and some appeared to compensate with strong involvement in care. Others still seemed to rely on gossip. The young assistants kept in the background at most times.

Consistent with other research (Krogstad, Hofoss,Veenstra, & Hjortdal, 2006), the head nurse had a decisive role in this facility, and her balanced ideology seemed to allow for a wide specter of performances; from high quality care to accidental and incongruent care. Some staff appeared competent to balance routines and procedures with on the spur-of-the-moment actions when caring for the new residents. This is different from other studies (Wiersma, 2010, Harnett, 2010) where routines are regarded as both rigid and adhered to as goals within themselves. According to Berger and Luckmann (1966, 2006), habits and routines may be understood as patterns of actions alternating between individual performance and social control. Routines contribute to predictability and habits in one's work. This may liberate staff to be flexible and creative in interactions. Some appreciated developing their work in close interaction with the new resident, and quickly adapted to specific situations. In a transition perspective, Schumacher et al. (1999) underscore that the goal of nursing is the creation of an environment that is dynamic and flexible enough to change in synchrony with the older residents’ evolving needs. The findings show that some exploited situations and circumstances to facilitate the new residents’ feeling connected, being situated, and developing confidence and mastery which are in line with Meleis et al.'s (2000) process indicators moving a resident toward a healthy transition. Yet these actions were seldom appreciated and shared in formal settings, and were frequently referred to as the “little things that matter.” This demonstrates the taken-for-granted in this particular practice situation. But as MacLeod (1994) states, these nursing practices are often purposeful, complex, multifaceted, and patient centered. Research on these kind of practices related to transition would allow more insight into process indicators that facilitate healthy transitions, and are therefore of great significance. Juritzen and Heggen (2009) concluded in their study that little of the nurses’ work is written down and that a lot may not even be verbalized.

Furthermore, our study found that most of what was shared during oral shift reports initially concerned medical matters. Meleis et al. (2000) argue that actions facilitating a healthy transition transcend biomedical driven strategies and focus on residents’ “lived experiences, the daily life events, and life-styles” (p. 70). These aspects were mainly shared in informal staff interactions. One drawback with these settings is that they do not include everybody, and may thereby have contributed to coincidental actions or lack of actions. Although this applied to all staff, particularly part-time unlicensed staff could miss vital information about how to assist the new residents. Our study shows that all residents, regardless of cognitive function, at times could be ignored. This is different from Slettebø et al. (2010) who found that particularly the cognitively impaired were prone to experiencing unjust health-services due to contextual restraints.

The findings show multiple staff perspectives concerning the new residents’ self-care capabilities. Most residents had experienced multiple transitions before they finally moved into a private room in the LTCF (Eika et al., 2014). Hence their self-care capabilities had been challenged for some time. After admission, although some primary auxiliaries and nurses focused on the residents’ maintenance of their self-care, others ignored it blaming it on their own personal qualities, embedded beliefs, and work circumstances. Staff, regardless of professional background, did not seem preoccupied with soliciting knowledge about the residents’ past self-care practices from their home environment. Jensen and Cohen Mansfield (2006) found in their study that this applied to hands-on nursing assistants who were almost totally lacking in the knowledge of residents’ previous self-care routines. Most residents in our study came from other health care settings and not their homes, and staff focused mainly on information from these settings which they often did not find helpful.

Local affinity influenced the staff's actions. Staff involvement in rural contexts has been observed in other studies (Congdon & Magilvy, 1995/2007, Ryan & McKenna, 2013). Ryan and McKenna (2013) found that when the older residents and staff knew each other and friends and neighbors visited, this facilitated a more positive transition for older people and their families. Many studies show that relationships with peer residents (Bradshaw, Playford, & Riaz, 2012) and staff (Glover, 2001, Coughlan & Ward, 2007, Nakrem, Vinsnes, Harkless, Paulsen, & Seim, 2011) contribute greatly to the residents’ experiences of good quality life in nursing homes. Furthermore, familiarity with people and places helped the residents still feel part of their community after admission. Ytrehus (2004) found in her study of younger older people's reflections about moving that familiar places played a more significant role than did their house in their experiences of continuity and connection. Still our findings show that the influence of local community could lead to stigmatization of some residents and sometimes overshadow their complex needs. Staff in some instances appeared to mix professional roles and local roles, whereas at other times they tried to maintain the boundary between their professional role and the role as local persons. Masvie and Ytrehus (2013) also found in their study of mental health workers’ experiences in small municipalities that their professional roles could affect their private life and the role and relations they had in the community as a citizen.

### 

#### Strengths and limitations of the study

The study is limited to one LTCF in a nursing home in rural Norway. The strength of this study is the focus on different staff's action in this period of change for older people, and a multiple methods approach in data collection. The findings are not altogether transferable to other similar settings due mainly to the lack of similar research to compare with.

## Conclusion

Nursing staff's actions varied from moving the new residents in the direction of health to moving them toward vulnerability and risk. Some powerful influential forces on staff's actions during these times were the head nurse's leadership style; individual staff's formal position, traits, and enthusiasm; resident and staff mix; and local transparency. This study gives a picture of different staff's actions at a key point in the residents’ ongoing transition process, which shows that both unlicensed and licensed staff were susceptible to performing congruent as well as incongruent care. Our study contributes with new knowledge describing circumstances and mechanisms played out among staff within the LTCF setting which goes beyond the fact that they were trained in health care or not. Currently recruitment of licensed personnel in the care of the elderly is a challenge in many countries. The Norwegian Research Council, in line with Report to the Storting No. 13, underscores the need for research exploring different professional groups’ cooperation and interaction skills on both individual and organizational levels. Additional studies need to explore this issue further.
